# Peroxisome Proliferator-Activated Receptor-γ Modulates the Response of Macrophages to Lipopolysaccharide and Glucocorticoids

**DOI:** 10.3389/fimmu.2018.00893

**Published:** 2018-05-08

**Authors:** Michael Heming, Sandra Gran, Saskia-L. Jauch, Lena Fischer-Riepe, Antonella Russo, Luisa Klotz, Sven Hermann, Michael Schäfers, Johannes Roth, Katarzyna Barczyk-Kahlert

**Affiliations:** ^1^Institute of Immunology, University of Muenster, Muenster, Germany; ^2^Department of Neurology, University of Muenster, Muenster, Germany; ^3^European Institute for Molecular Imaging, University of Muenster, Muenster, Germany

**Keywords:** PPARgamma, glucocorticoids, macrophage, migration, anti-inflammatory

## Abstract

Although glucocorticoids (GC) represent the most frequently used immunosuppressive drugs, their effects are still not well understood. In our previous studies, we have shown that treatment of monocytes with GC does not cause a global suppression of monocytic effector functions, but rather induces differentiation of a specific anti-inflammatory phenotype. The anti-inflammatory role of peroxisome proliferator-activated receptor (PPAR)-γ has been extensively studied during recent years. However, a relationship between GC treatment and PPAR-γ expression in macrophages has not been investigated so far. Studies using PPAR-γ-deficient mice have frequently provided controversial results. A potential reason is the use of primary cells, which commonly represent inhomogeneous populations burdened with side effects and influenced by bystander cells. To overcome this constraint, we established ER-Hoxb8-immortalized bone marrow-derived macrophages from *Pparg^fl/fl^* and *LysM-Cre Pparg^fl/fl^* mice in this study. In contrast to primary macrophages, the ER-Hoxb8 system allows the generation of a homogeneous and well-defined population of resting macrophages. We could show that the loss of PPAR-γ resulted in delayed kinetic of differentiation of monocytes into macrophages as assessed by reduced F4/80, but increased Ly6C expression in early phases of differentiation. As expected, PPAR-γ-deficient macrophages displayed an increased pro-inflammatory phenotype upon long-term LPS stimulation characterized by an elevated production of pro-inflammatory cytokines TNF-α, IL1-β, IL-6, IL-12 and a reduced production of anti-inflammatory cytokine IL-10 compared to PPAR-γ WT cells. Moreover, PPAR-γ-deficient macrophages showed impaired phagocytosis. GC treatment of macrophages led to the upregulation of PPAR-γ expression. However, there were no differences in GC-induced suppression of cytokines between both cell types, implicating a PPAR-γ-independent mechanism. Intriguingly, GC treatment resulted in an increased *in vitro* migration only in PPAR-γ-deficient macrophages. Performing a newly developed *in vivo* cell-tracking experiment, we could confirm that GC induces an increased recruitment of PPAR-γ KO, but not PPAR-γ WT macrophages to the site of inflammation. Our findings suggest a specific effect of PPAR-γ on GC-induced migration in macrophages. In conclusion, we could demonstrate that PPAR-γ exerts anti-inflammatory activities and shapes macrophage functions. Moreover, we identified a molecular link between GC and PPAR-γ and could show for the first time that PPAR-γ modulates GC-induced migration in macrophages.

## Introduction

Peroxisome proliferator-activated receptors (PPARs) are members of the nuclear receptor superfamily containing three isoforms: PPAR-α, PPAR-β, and PPAR-γ. PPAR-γ was originally characterized as a regulator of fatty acid synthesis, glucose metabolism, and is a known factor promoting differentiation of adipocytes (1, 2). Due to its involvement in regulation of many physiological processes such as lipid metabolism, response to insulin, and proliferation, PPAR-γ became an attractive therapeutic target for the treatment of metabolic disorders (1, 3).

Over time, it became more and more evident that PPAR-γ also plays a pivotal role in the immune system (4). PPAR-γ is expressed on numerous cells of the immune system, including monocytes/macrophages, dendritic cells (DCs), T and B lymphocytes, and platelets (5–12). Besides being the master regulator of adipocyte differentiation, PPAR-γ is also induced during differentiation of monocytes to macrophages (13, 14). However, PPAR-γ-deficient embryonic stem cells were shown to be capable of differentiating to macrophages (15). A recent study demonstrated that PPAR-γ determines the differentiation of fetal monocytes into alveolar macrophages, while being dispensable for macrophage differentiation of other organs (16).

Moreover, PPAR-γ exerts anti-inflammatory properties that can modulate the immune inflammatory response. It has been shown that PPAR-γ agonists act as negative regulators of monocytes and macrophages and dose-dependently inhibit the production of pro-inflammatory cytokines, such as TNF-α, IL-1β, and IL-6, in human monocytes (17, 18). However, some studies have challenged the anti-inflammatory activity of PPAR-γ agonists (7, 19, 20). It has been shown that the PPAR-γ agonist rosiglitazone suppresses the LPS-induced expression of pro-inflammatory cytokines PPAR-γ-dependently at low concentrations, but PPAR-γ-independently at high concentrations (7). Therefore, the use of a PPAR-γ KO model is essential to gain a better insight into the effects of PPAR-γ on inflammation.

Peroxisome proliferator-activated receptor-γ has also been reported to participate in controlling an alternative activation of monocytes and macrophages (21). A positive correlation between the expression of M2 markers and PPAR-γ was observed in human atherosclerotic lesions: PPAR-γ activation of human primary monocytes skewed them toward an anti-inflammatory M2 phenotype (22). This observation was further supported by the finding that Th2 cytokines like IL-4 and IL-13 enhance PPAR-γ expression in monocytes/macrophages and that IL-4-STAT6-PPAR-γ signaling axis in monocytes is crucial for their differentiation into alternatively activated macrophages (23–27). Recent studies suggested a central role of PPAR-γ in the resolution of inflammation. While the loss of PPAR-γ resulted in a defective resolution of inflammation with chronic leukocyte recruitment, PPAR-γ agonists promoted the cessation of neutrophil recruitment and thus favored the resolution of inflammation (5).

Activation of PPAR-γ can elicit different effects, depending on the nature of activating factor. Exposure of macrophages to oxLDL has been reported to activate PPAR-γ in a PKC-dependent manner that in consequence led to an enhanced expression of CD36 and further stimulation of oxLDL uptake (28, 29). Besides, it was demonstrated that PPAR-γ antagonist decreased the phagocytic capacity of macrophages by inhibiting the expression of molecules pivotal for the recognition of apoptotic cells (30). Consistently, it was shown that skin wound healing was impaired in PPAR-γ-deficient mice because of defective clearance of apoptotic cells (31). On the other hand, it was observed that PPAR-γ-deficient inflammatory monocytes displayed enhanced recruitment to the site of infection (32).

Glucocorticoids (GC) represent the most widely and frequently used anti-inflammatory drugs (33). However, prolonged therapy leads to deleterious side effects, which frequently limit their clinical use (33). GC affect nearly all the cells of the immune system, but there is growing evidence for cell-type specific mechanism. However, the exact effect of GC on monocytes and macrophages is still not well defined. Our previous studies have shown that the treatment of naïve monocytes with GC did not lead to a global suppression of their function but rather induced differentiation of cells with an anti-inflammatory and regulatory phenotype (34, 35). Furthermore, GC promoted survival of this anti-inflammatory phenotype in macrophages (36). However, a functional relationship between GC treatment and PPAR-γ expression in macrophages has not been investigated so far.

Our previous genome-wide expression studies have shown that one of the nuclear factors significantly upregulated by GC in macrophages is PPAR-γ. However, the influence of PPAR-γ on GC-induced phenotype in monocytes and macrophages has not yet been addressed. In the present study, we investigated for the first time the role of PPAR-γ in transiently immortalized bone marrow-derived macrophages from *LysM-Cre Pparg^fl/fl^* and *Pparg^fl/fl^* mice.

## Materials and Methods

### Generation of PPAR-γ-Deficient ER-Hoxb8 Cells

*Pparg^fl/fl^* (PPAR-γ WT) and *LysM-Cre Pparg^fl/fl^* (PPAR-γ KO) mice were kindly provided by the Department of Neurology in Münster, Germany. ER-Hoxb8 cells were generated as described previously (37). Briefly, bone marrow cells (BMCs) were isolated from the femurs and tibiae. Progenitor cells were resuspended in Iscove’s Modified Dulbecco’s Medium (StemCell Technologies, Cologne, Germany), containing 10% fetal bovine serum (FBS) (PAN-Biotech, Aidenbach, Germany), 1% P/S/G (Biochrome, Berlin, Germany) and supplemented with 10 ng/ml IL-3, 20 ng/ml IL-6 (Peprotech, Rocky Hill, CT, USA), and 1% SCF (CHO cells supernatant), and cultured for 48 h. The MSCV retroviral vector, expressing an estrogen dependent Hoxb8 transcription factor (ER-Hoxb8), was kindly provided by the laboratory of Georg Haecker (University Hospital of Freiburg, Germany). Directly before virus transfection, cells were harvested, washed, and resuspended in complete RPMI 1640 medium (Biochrome, Berlin, Germany) supplemented with 1 µM β-estradiol (Sigma-Aldrich, Darmstadt, Germany) and 40 ng/ml GM-CSF (Immunotools, Friesoythe, Germany). 1 × 10^6^ cells were subjected to spinoculation with 1 ml of MSCV retroviral vector in the presence of Lipofectamine (Thermo Fischer Scientific, Waltham, MA, USA) and incubated for 24 h. Afterward, infected cells were cultured in RPMI 1640 supplemented with 10% FBS, 1% P/S/G, 40 ng/ml GM-CSF, and 1 µM β -estradiol. Cells were split every 2–3 days in densities of 1–3 × 10^5^ cells/well into a new six-well plate. This procedure was continued for over 3 weeks to produce immortalized macrophage progenitor lines.

### ER-Hoxb8 Cell Culture

ER-Hoxb8 cells were cultured in tissue culture plates in RPMI 1640 medium supplemented with 10% FBS, 1% P/S/G, 2% GM-CSF (B16 cells supernatant), and 1 µM β-estradiol (Sigma-Aldrich, Steinheim, Germany). Cells were split every 3 days.

### Differentiation of ER-Hoxb8 Cells

ER-Hoxb8 cells were washed three times with PBS/1% FBS to completely remove β-estradiol. Subsequently, 2 × 10^6^ PPAR-γ WT and 1 × 10^6^ PPAR-γ KO ER-Hoxb8 cells were seeded in 15 ml medium without β-estradiol per dish in untreated Petri dishes. Cells were differentiated for 2–5 days. Non-adherent cells were aspirated and discarded. To detach the adherent macrophages, ice-cold PBS supplemented with 10 mM EDTA (Roth, Karlsruhe, Germany) was used. Cells were incubated on ice for 10 min and vigorously pipetted up and down.

### Stimulation of Differentiated Cells

1 × 10^6^ ER-Hoxb8 macrophages were plated in 1 ml medium per well in non-treated six-well plates. The cells were stimulated with dexamethasone (DEX) (100 nM) and/or LPS (1 µg/ml) (both from Sigma-Aldrich, Steinheim, Germany) for 3, 24, and 48 h or left untreated.

### Isolation of Bone Marrow-Derived Monocytes

Monocytes were purified from bone marrow cells isolated from murine femur and tibiae. Erythrocytes were depleted by osmotic shock, cells were washed and collected by centrifugation. Subsequently, cells were separated using a Ficoll gradient centrifugation and the cells in the interphase were collected. Monocytes were isolated by negative selection and T cells, B cells, and DCs were removed using magnetic beads coupled to anti-CD90, anti-CD19, and anti-CD11c antibodies and using MACS technology. Finally, cells were resuspended in Dulbecco’s Modified Eagle Medium (DMEM) supplemented with 10% fetal bovine serum (FBS), 1% penicillin-streptomycin-L-glutamine (Biochrome, Berlin, Germany) and 20% L929 cell supernatant. Monocytes were cultured for 24 h and/or 48 h in the presence of 1 µM β-estradiol or left untreated. Subsequently cell lysates were prepared or the cells were harvested, washed twice to deprive of b-estradiol (Sigma-Aldrich, Steinheim, Germany) and stimulated with 100 nM dexamethasone (from Sigma-Aldrich, Steinheim, Germany) or ethanol (EtOH) as control. After 24 h cells were harvested and RNA was isolated.

### Quantitative PCR

RNA isolation was performed using the NucleoSpin RNA II Kit (Macherey-Nagel, Düren, Germany) according to the manufacturer’s protocol. RNA concentration was measured with Nanodrop (Paqlab, Erlangen, Germany). 1 µg RNA was used to synthesize cDNA with RevertAid H Minus Reverse Transcriptase (Thermo Scientific, Waltham, MA, USA). qPCR was performed using the KAPA SYBR FAST qPCR Kit with *Rpl* as the housekeeping gene. The following primers were used: *C5ar1* forward 5′-GGA ATG GTT TTG AAT TTC CTG GTC A-3′, *C5ar1* reverse 5′-AGA CAG TCT CCC TGG GTA AGC-3′; *Ccr2* forward 5′-TGA TAG TAT GCC GTG GAT GAA CTG-3′, *Ccr2* reverse 5′-TGC AAG TTC AGC TGC CTG C-3′; *Cd14* forward 5′-TTT GCA TCC TCC TGG TTT CTG A-3′, *Cd14* reverse 5′-GCT TTT ACC CAC TGA ACC ATC TTG-3′; *Cd38* forward 5′-TGC CCA CAT TGG AGT GAA AAC T-3′, *Cd38* reverse 5′-ACC CAT TGA GCA TCA CTT GGA C-3′; *Cd86* forward 5′-ACC TCG GTG CTC AAC AGG TA-3′, *Cd86* reverse 5′-TTT CCC TCC TTC CAC ACA AGC-3′; *Il1b* forward 5′-TGT CTT GGC CGA GGA CTA AGG-3′, *Il1b* reverse 5′-TGG GCT GGA CTG TTT CTA ATG C-3′; *Il6* forward 5′-TGA GAT CTA CTC GGC AAA CCT AGT G-3′, *Il6* reverse 5′-CTT CGT AGA GAA CAA CAT AAG TCA GAT ACC-3′; *Il10* forward 5′-GGG TTG CCA AGC CTT ATC G-3′, *Il10* reverse 5′-TCT CAC CCA GGG AAT TCA AAT G-3′; *Il12b* forward 5′-CCA AGT GGA ATG CTA GAA TAT CTA TGC-3′, *Il12b* reverse 5′-GCC TGT TAC ACT CAA GGT GAT GTG-3′; *Nos2* forward 5′-CCT CAT TGG CCA GCT GCT T-3′, *Nos2* reverse 5′-GGT CCG CAA GAG AGT GCT GTT-3′; *Rpl* forward 5′-TGG TCC CTG CTG CTC TCA AG-3′, *Rpl* reverse 5′-GGC CTT TTC CTT CCG TTT CTC-3′; *Tnfa* forward 5′-AGA AAC ACA AGA TGC TGG GAC AGT-3′, *Tnfa* reverse 5′-CCT TTG CAG AAC TCA GGA ATG G-3′; *Tlr4* forward 5′-GAA CAA GAA ACG GCA ACT TGG AC-3*′, Tlr4* reverse 5′-TAC CCC TGG AAA GGA AGG TGT C-3′.

### Western Blot

Cells were lysed using lysis buffer (1% NP-40, 20 mM HEPES, 350 mM NaCl, 20% glycerin, 1 mM glycerin, 1 mM MgCl_2_, 0,5 mM EDTA, 0,1 mM EGTA and protease inhibitor) and protein concentration was determined. Equal amounts of proteins were separated by SDS-PAGE (about 50 µg protein per lane) and subsequently blotted on nitrocellulose membrane. The membrane was incubated with an antibody against PPAR-γ (C26H12, Cell Signaling Technology, Danvers, MA, USA), a rabbit polyclonal antibody against glucocorticoid receptor (M-20, Santa Cruz Biotechenology, Dallas, US) and β-Actin (Sigma-Aldrich, Steinheim, Germany) followed by a HRP-linked secondary antibody (Dako, Santa Clara, CA, USA). Chemiluminescence signal was detected using ChemiDoc XRS+ (Bio-Rad, Munich, Germany). To quantify the signal intensity, ImageJ (National Institutes of Health) was used.

### Measurement of Cytokine Production

The amount of IL-1β in cell supernatants was determined using the Mouse IL-1 beta ELISA Ready-SET-Go! (eBioscience, Frankfurt, Germany) according to manufacturer’s instructions. Secreted IL-10, IL-12p70, and CCL5 in cell supernatants were quantified using the BD CBA Flex Set (BD Biosciences, Heidelberg, Germany). The analysis was carried out with FACSCalibur and FCAP Array software (BD Biosciences, Heidelberg, Germany).

### Flow Cytometry

For the detection of cell surface molecules, cells were incubated with the following antibodies: CD14 APC (eBioscience, Frankfurt, Germany), CD36 PE, CD38 PE, CD86 FITC, F4/80 APC, Ly6C FITC (all from BioLegend, Fell, Germany), or the appropriate isotype controls. Flow cytometric analysis was performed using FACSCalibur and analyzed using FlowJo software (Tree Star, Ashland, OR, USA).

### Chromatin Immunoprecipitation (ChIP)

#### Cell Fixation and Shearing

ER-Hoxb8 cells were differentiated for 2 days as described above and fixed using 1% formaldehyde (Sigma-Aldrich, St. Louis, USA) for 10 min. Reaction was stopped by addition of glycine to a final concentration of 0.125 M. Adherent cells were gently scraped, washed twice, and lysed. Cell lysates were sonicated using a Branson Sonifier 250 Analog Ultrasonic Homogenizer (Labequip, Markham, ON, Canada) with 50% duty cycle for 10 rounds, each round with 30 s pulses, and with 1-min pauses on ice. Cell lysates were centrifuged (1 min, 8,000 *g*, 4°C), cell debris was discarded and supernatants were frozen at −20°C for subsequent immunoprecipitation.

#### Preparation of Input DNA

To generate input DNA, 10% of lysate was filled up with ddH_2_O up to 500 µl and 10 µl 5 M NaCl, 10 µl EDTA (both from Active Motif, Carlsbad, MA, USA), 20 µl Tris pH 6.5, 100 µg proteinase K (AppliChem, Darmstadt, Germany), and 1.5 µg RNAse A (Sigma-Aldrich, St. Louis, MO, USA) were added and samples were incubated for 5 h at 65°C to reverse crosslink and digest proteins/RNA. DNA was isolated by phenol–chloroform extraction and the concentration was measured using a spectrophotometer (peQLab, Erlangen, Germany) and chromatin shearing efficiency was examined via agarose gel electrophoresis.

#### Immunoprecipitation

24 µg DNA-equivalent chromatin was used per immunoprecipitation. The DNA–protein complexes were immunoprecipitated with 3 µg of mouse monoclonal antibody against PPAR-γ antibody [ab41928] (Abcam, Cambridge, UK). ChIP was performed with ChIP-IT^®^ protein G magnetic beads as described in ChIP-IT^®^ Express Enzymatic kit (Active Motif, Carlsbad, MA, USA).

#### Preparation of Sample DNA

Eluted chromatin was mixed with 10 µl 5 M NaCl, 10 µl EDTA, 20 µl Tris pH 6.5, 100 µg proteinase K and incubated for 5 h at 65°C to reverse crosslink between DNA and protein. DNA was isolated using phenol–chloroform extraction as described above and subsequently used as PCR templates. The DNA was amplified with the primers flanking putative PPAR response element (PPRE) (+705 to +717) in the intron 1 of the mouse *Cd38* gene: forward 5′-*GCCACAGCCATGCTTCTGG*-3′ and reverse: 5′-*CCCCACAGCAAGCTGAGCA-*3′ (38). qPCR was performed using the KAPA SYBR FAST qPCR Kit (Sigma-Aldrich, St. Louis, MO, USA). For the visualization of the DNA bands, agarose gel electrophoresis was performed.

### Functional Assays

#### Phagocytosis of Latex Beads

Prior to use, FluoSpheres polystrene microspheres (Thermo Scientific, Waltham, MA, USA) were shortly incubated in a bath sonicator to break down any aggregates. Subsequently, cells were incubated with latex beads at a ratio 1:10 for 2 h. The rate of phagocytosis was determined by flow cytometry using FACSCalibur.

#### Oxidative Burst

Cells were stimulated with 10 nM PMA (Abcam, Cambridge, UK) or left untreated. After an incubation time of 15 min, 15 µM DHR 123 (Sigma-Aldrich, Steinheim, Germany) was added for another 15 min. Next, the immunofluorescence signal was analyzed using FACSCalibur.

#### Nitric Oxide (NO) Assay

As an indicator of NO production, the amount of nitrite in culture supernatant was measured using Griess Reagent. Briefly, 400 µl culture supernatant was mixed with 400 µl Griess reagent and absorption was measured at 560 nm in the microplate reader. The quantification of nitrite was determined from a sodium nitrite standard curve.

#### Transmigration Assay

Cell migration assay was performed using transwell filters with 5-µm pore size (Corning, Wiesbaden, Germany). 600 µl medium with or without 100 ng/ml C5a (R&D Systems, Wiesbaden, Germany) was filled in each well. Filters were inserted and 1 × 10^6^ cells in 100 µl medium were added to the upper chamber. Cells were allowed to transmigrate for 4 h. Migrating cells found in the lower chamber were harvested and counted.

### *In Vivo* Migration Assay

A cutaneous granuloma model (CG) was established by subcutaneous injection of 200 µl BioGel P-100 (Bio-Rad, Munich, Germany), containing 20 µg LPS/200 μl BioGel (left plug) or BioGel only (right plug, control) at the dorsal flank region of mice. 24 h later differentiated ER-Hoxb8 macrophages were labeled with the fluorescent dye DiD (Thermo Scientific, Waltham, MA, USA) by incubating 1 × 10^6^ cells/ml with 47.5 µl DiD for 5 min. Afterward, cells were washed twice. 5 × 10^6^ DiD labeled cells in 200 µl PBS were injected in the tail vein of CG mice. Labeled cell migration was tracked 3, 6, 24, 30, and 48 h post injection (p.i.) by using IVIS system (Perkin Elmer, Waltham, MA, USA) and an appropriate filter setting of 605/680 nm (excitation/emission). For data correction, a baseline scan directly before cell application was performed. During measurements mice were kept warm and under anesthesia with 2% isoflurane (Dräger, Lübeck, Germany). For data calculation, regions of interest were located around the plugs with Living Image Software to determine average radiant efficiency. Values were then corrected for background signal by subtracting baseline measurements and labeling efficiency by normalizing to WT control cells due to dilution series of labeled cells. Data were presented as fluorescence intensity. For image display, color scales were normalized to WT control cells due to the labeling efficiency by adapting Min/Max threshold.

### Study Approval

All animal experiments were carried out in accordance with the German animal protection law (TierSchG). The protocol was approved by the government authorities (Landesamt für Natur, Umwelt und Verbraucherschutz Nordrhein-Westfalen).

### Statistical Analysis

The statistical significance of the data was determined using two-way ANOVA with Bonferroni’s *post hoc* multi-comparisons with GraphPad Prism (GraphPad, San Diego, CA, USA). A probability (*p* value) of <0.05 was considered significant. *p* < 0.05 is denoted by *, *p* < 0.01 by **, and *p* < 0.001 by ***. Error bars show the SEM.

## Results

### DEX Induces the Upregulation of PPAR-γ

**Figure 1:**
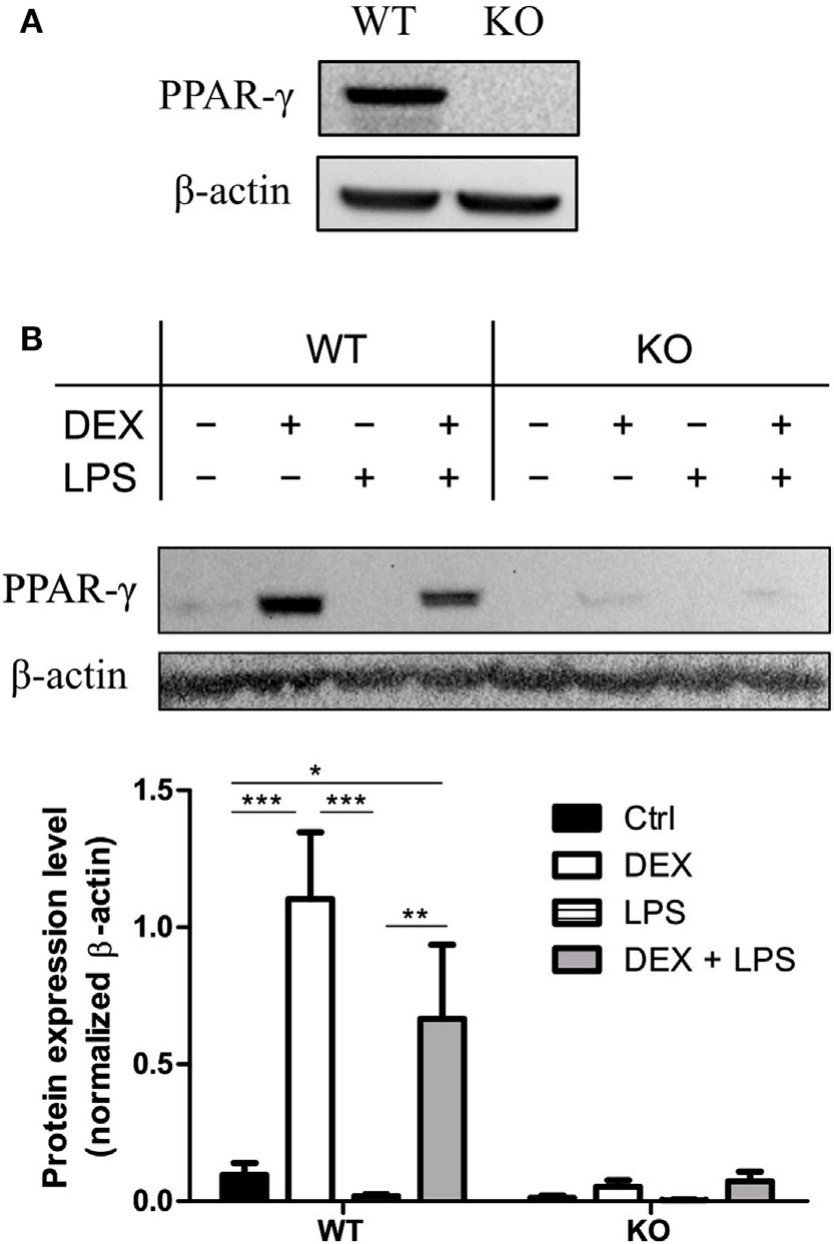
Dexamethasone (DEX) stimulation leads to the upregulation of peroxisome proliferator-activated receptor (PPAR)-γ. **(A)** Western blot of PPAR-γ was performed with ER-Hoxb8 macrophage progenitor cells stimulated with DEX for 24 h. β-Actin was used as a loading control. **(B)** ER-Hoxb8 cells were differentiated to macrophages for 5 days and stimulated with DEX (100 nM) and/or LPS (1 µg/ml) for 24 h. The upper part displays an immunoblot from one representative experiment including the β-Actin loading control. The lower part shows the quantification of western blot results from three independent experiments. The signal intensity of PPAR-γ was divided by the signal intensity of β-Actin, which served as a loading control. **(A,B)** The bars represent the mean with the SEM of three independent experiments (*p < 0.05, **p < 0.01, ***p < 0.001) (calculated by using two-way ANOVA).

To test whether immortalization of the BM progenitor cells has an influence on PPAR-γ expression, we analyzed the expression of PPAR-γ in PPAR-γ WT and PPAR-γ KO ER-Hoxb8 macrophage progenitors. We could easily detect a mRNA coding for *Pparg* in WT, but not in PPAR-γ KO, progenitor cells (data not shown). However, the PPAR-γ protein expression in unstimulated progenitor cells was under the detection limit in Western blotting. In contrast, we observed a clear expression of PPAR-γ by Western Blot after prestimulation of WT progenitor cells for 24 h with DEX which was not observed in PPAR-γ KO cells (Figure 1A). Next, we differentiated ER-Hoxb8 progenitor cells for 5 days to macrophages (d5) and subsequently stimulated them with DEX (100 nM) and/or LPS (1 µg/ml) for 24 h. Expression of transcript coding for *Pparg* and the PPAR-γ protein were determined using qPCR and Western blot, respectively. Differentiation of progenitor cells to macrophages was accompanied by slight increase in PPAR-γ expression in WT ER-Hoxb8 macrophages as compared to progenitor cells (a weak band corresponding to PPAR-γ could be detected in unstimulated d5 macrophages). LPS-treated WT macrophages showed no PPAR-γ expression. However, there was a significant upregulation of PPAR-γ upon stimulation with DEX and combination of DEX + LPS in WT macrophages (Figure 1B). To exclude the influence of β-estradiol on GC sensitivity, we prepared bone marrow-derived monocytes and pre-cultured them in the presence of β-estradiol for 24 and 48 h. Subsequently, we performed Western blot analysis of the glucocorticoid receptor (GR) expression in cell lysates. There were no differences in GR expression between control and β-estradiol-treated cells (Data Sheet 1 in Supplementary Material). Additionally, we stimulated control and β-estradiol-treated bone marrow-derived monocytes with DEX for 24 h and subsequently analyzed the expression of GC-regulated genes: *Cd163, Cd121b, Cd38, Mrc1*, and *Il10*. All these molecules were upregulated to a similar extent in the control and β-estradiol-treated cells (Data Sheet 1 in Supplementary Material).

### Delayed Differentiation Kinetics of PPAR-γ-Deficient Macrophages

**Figure 2:**
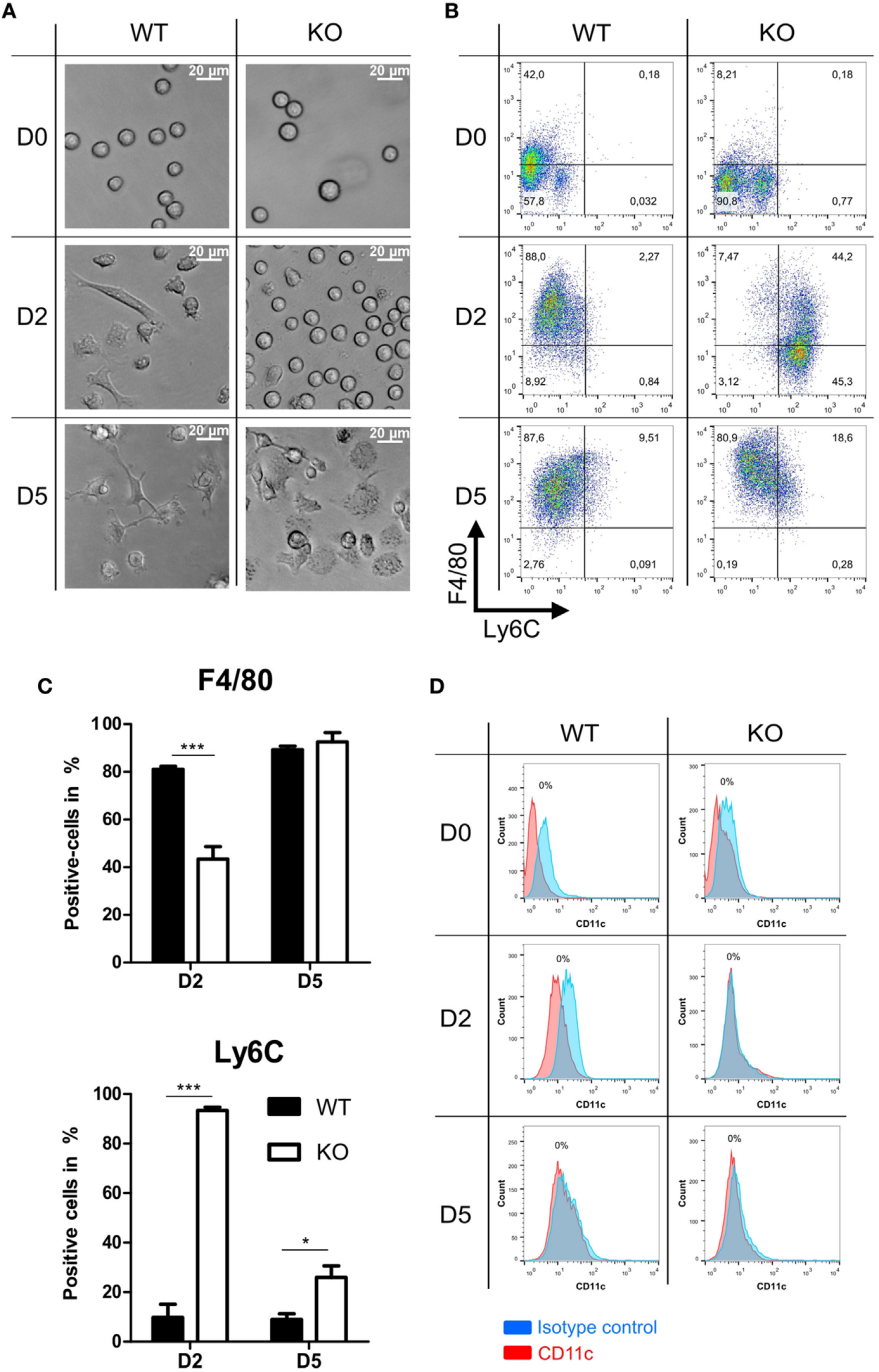
Differentiation of peroxisome proliferator-activated receptor-γ-deficient cells shows delayed kinetics. **(A)** On day 0, 2, and 5 of differentiation, ER-Hoxb8 pool cells were analyzed using light microscopy. A result from one representative experiment out of three performed is shown. **(B,C)** The expression of Ly6C and F4/80 on day 0, 2, and 5 of differentiation was analyzed using FACS. Dot plots show the results from one representative experiment. **(D)** The expression of CD11c was investigated using FACS. The bars represent the mean with the SEM of three independent experiments (*p < 0.05, ***p < 0.001) (calculated by using two-way ANOVA).

Further we analyzed morphology and differentiation kinetics of ER-Hoxb8 progenitor cells in more detail. WT and PPAR-γ KO ER-Hoxb8 cells were differentiated to macrophages in the presence of GM-CSF. The treatment of progenitor cells with GM-CSF did not lead to the differentiation of DCs as assessed by the expression of DC marker CD11c (Figure 2D). The morphology of differentiating macrophages was assessed under the light microscope and expression of Ly6C and macrophage marker F4/80 was determined in flow cytometry. There were no morphological differences observed between WT and PPAR-γ KO progenitor cells (Figure 2A upper panel). However, progenitor cells differentiated to macrophages with different kinetics. Already after 2 days of differentiation, WT cells became adherent and displayed an irregular cell shape characteristic for cells undergoing differentiation. In contrast, at this time point, PPAR-γ-deficient cells still showed the regular, round shape similar to that observed on day 0 (Figure 2A middle panel). Moreover, we still detected strong proliferation of PPAR-γ KO cells, despite deprivation of estradiol from culture medium, which regulates transcriptional function of ER-Hoxb8 and thus sustains cell proliferation. On day 5 of differentiation, WT cells displayed a typical macrophage morphology, characterized by cell surface ruffles and lamellipodia, strong adherence, reduction in the nucleocytoplasmic ratio, and enhanced granularity. In contrast, PPAR-γ KO cells represented a mixed population of differentiated macrophages and immature cells (Figure 2A lower panel). Delayed differentiation kinetics of PPAR-γ KO cells could be further supported by flow cytometric analysis of Ly6C and F4/80 in the course of differentiation. On day 2 of differentiation, the majority of WT cells did not express Ly6C, but were positive for F4/80, whereas the majority of PPAR-γ KO cells were Ly6C positive, but co-expressed F4/80 on approximately only 40% of cells (Figures 2B,C). However, on day 5 of differentiation, over 50% of the PPAR-γ KO macrophages lost Ly6C expression, while almost all WT and PPAR-γ KO macrophages expressed F4/80, indicating a catch-up of differentiation in PPAR-γ KO cells (Figures 2B,C). No difference was observed in the expression of CD11b between both cell types on day 2 and day 5 of differentiation (data not shown).

### Pro-Inflammatory Phenotype in PPAR-γ-Deficient Macrophages

**Figure 3:**
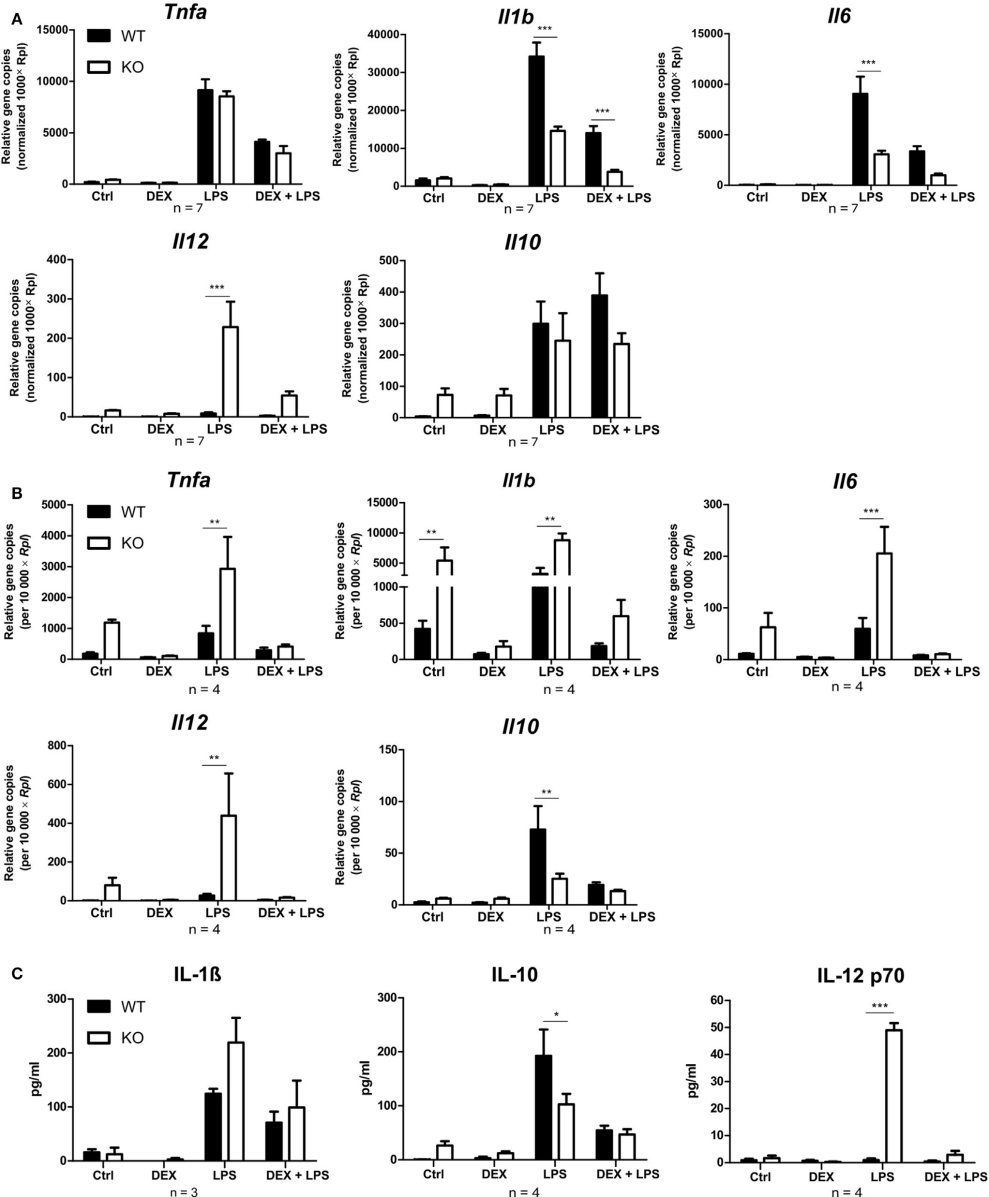
Peroxisome proliferator-activated receptor-γ KO cells display a pro-inflammatory phenotype. **(A–C)** ER-Hoxb8 cells d5 were treated with dexamethasone (DEX) (100 nM) and/or LPS (1 µg/ml) for 3 **(A)**, 24 **(B)**, or 48 h **(C)**. **(A,B)** The cells were analyzed for gene expression of the indicated genes using qPCR. **(C)** Supernatants were examined for IL-1β concentration using ELISA and for IL-10 and IL12 p70 concentration using CBA. **(A–C)** The bars represent the mean with the SEM of three to seven independent experiments (*p < 0.05, **p < 0.01, ***p < 0.001) (calculated by using two-way ANOVA).

**Figure 4:**
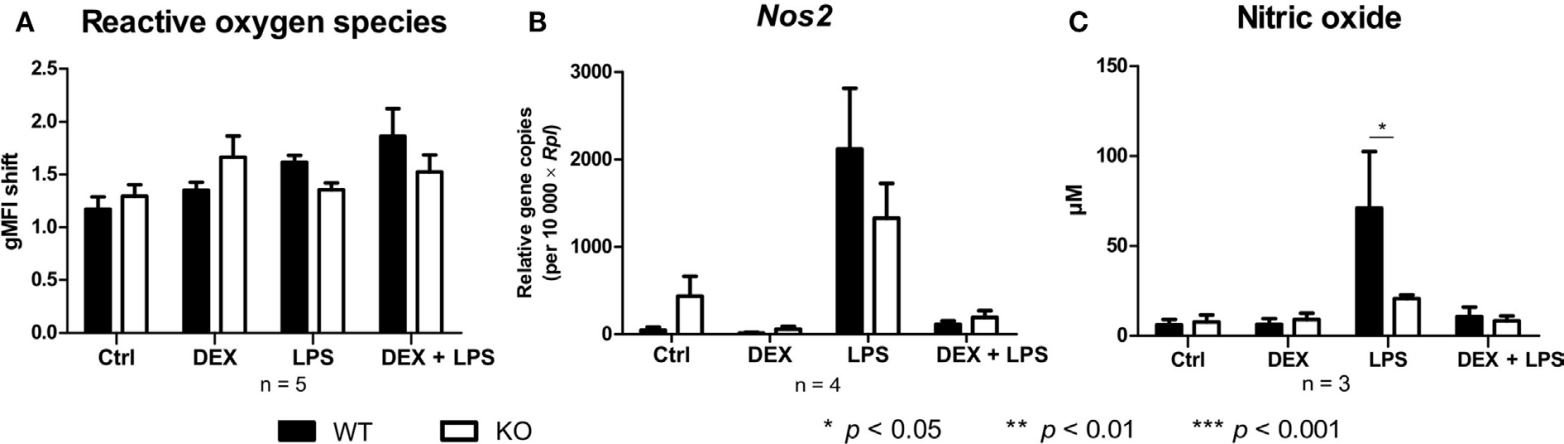
Peroxisome proliferator-activated receptor-γ deficiency influences nitric oxide (NO) but not reactive oxygen species production. **(A–C)** ER-Hoxb8 cells d5 were treated with dexamethasone (DEX) (100 nM) and/or LPS (1 µg/ml) for 48 h. **(A)** Untreated, DEX- and/or LPS-treated cells were stimulated with 10 nmol/l PMA for 15 min and oxidative burst was measured using DHR. The fluorescence of the cells was determined in FACS. **(B)** The cells were analyzed for the expression of *Nos2* using qPCR. **(C)** The production of NO was investigated by the NO assay. **(A–C)** The bars represent the mean with the SEM of three to five independent experiments (*p < 0.05, **p < 0.01, ***p < 0.001) (calculated by using two-way ANOVA).

Since PPAR-γ is known to exert anti-inflammatory properties in macrophages, we next analyzed the cytokine profile as well as the reactive oxygen species (ROS) and NO production of WT and PPAR-γ KO macrophages. WT and PPAR-γ KO macrophages d5 were stimulated with DEX (100 nM) and/or LPS (1 µg/ml) for 3 and 24 (qPCR) or 48 h (ELISA, CBA, ROS, and NO assay). During the early inflammatory response (3 h of LPS stimulation) significantly lower expression of *Il1b* and *Il6*, but no significant differences in the expression of *Tnfa* and *Il10*, were detected in PPAR-γ KO macrophages. However, PPAR-γ KO macrophages treated with LPS for 3 h displayed significantly elevated level of *Il12* (Figure 3A). In contrast, after prolonged stimulation with LPS (24 h) significantly higher levels of transcripts coding for the pro-inflammatory cytokines *Tnfa, Il1b, Il6*, and *Il12* was observed in PPAR-γ KO macrophages as compared to WT macrophages (Figure 3B). Interestingly, the expression of the anti-inflammatory cytokine *Il10* was reduced in LPS-stimulated PPAR-γ KO cells compared to WT cells (Figure 3B), implicating not a general overexpression of cytokines by PPAR-γ KO macrophages, but rather an inherent pro-inflammatory phenotype of these cells. However, DEX treatment of control and of LPS-stimulated macrophages led to a substantial inhibition of pro-inflammatory cytokine expression in both WT and PPAR-γ KO cells (Figures 3A,B). Subsequent analysis of cytokine secretion confirmed these differences. We detected comparable basal amounts of IL-1β, IL-12p70, and IL-10 in WT and PPAR-γ KO macrophages (Figure 3C). However, much higher concentrations of IL-1β and IL-12p70 and much lower concentrations of IL-10 could be measured in supernatants of LPS-stimulated PPAR-γ KO macrophages as compared to LPS-stimulated WT cells (Figure 3C). Analysis of the ROS production revealed no differences between WT and PPAR-γ KO cells (Figure 4A). Surprisingly, we observed reduced NO production in LPS-stimulated PPAR-γ KO macrophages as compared to WT cells (Figure 4C). Analysis of transcript encoding *Nos2* suggested a reduced *Nos2* expression in LPS-stimulated PPAR-γ-deficient cells, which explains diminished NO production in these cells (Figure 4B).

### PPAR-γ KO Macrophages Display Altered Expression of Cell Surface Proteins

**Figure 5:**
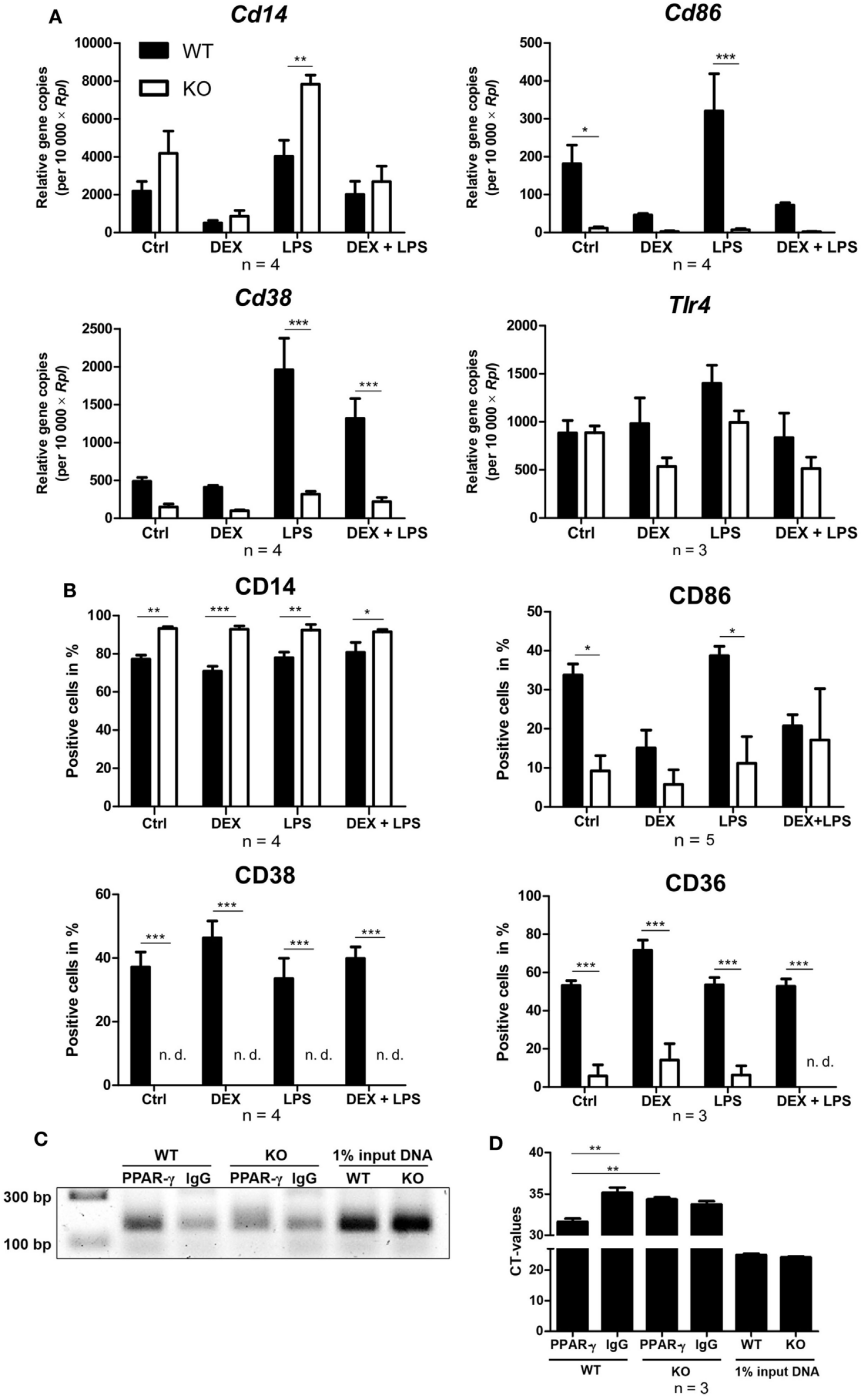
Altered expression of cell surface proteins on peroxisome proliferator-activated receptor (PPAR)-γ-deficient macrophages. **(A,B)** On day 5 of differentiation, ER-Hoxb8 cells were stimulated with dexamethasone (DEX) (100 nM) and/or LPS (1 µg/ml) for 24 **(A)** or 48 h **(B)**. **(A)** Gene expression of the indicated genes was analyzed using qPCR. **(B)** Surface expression of the indicated proteins was investigated by FACS. **(C,D)** On day 2 of differentiation, ER-Hoxb8 cells were subjected to chromatin immunoprecipitation to detect binding of PPAR-γ to putative PPAR response element located within intron 1 of murine CD38 gene. **(C)** Figure shows result from one representative experiment out of three performed. **(A,B,D)** The bars represent the mean with the SEM of three to five independent experiments (*p < 0.05, **p < 0.01, ***p < 0.001) (calculated by using two-way ANOVA).

Next, WT and PPAR-γ KO macrophages d5 were stimulated for 24 and 48 h with DEX and/or LPS and subsequently analyzed for the expression of different monocyte/macrophages markers in qPCR and flow cytometry, respectively. Analyzing the mRNA coding for *Cd14*, we observed a trend for enhanced expression of *Cd14* in PPAR-γ KO macrophages (Figure 5A). Flow cytometric analysis of CD14 surface expression revealed slightly upregulated expression of CD14 in PPAR-γ-deficient macrophages as compared to WT cells, which remained unaltered after DEX or/and LPS stimulation (Figure 5B). In contrast, we observed significantly reduced mRNA and protein expression of CD86 and CD38 in PPAR-γ KO macrophages (Figures 5A,B). Unstimulated WT and PPAR-γ KO macrophages did not shown any differences in *Tlr4* expression (Figure 5A). DEX and/or LPS-stimulated PPAR-γ KO macrophages revealed a modest yet not significant tendency to lower *Tlr4* expression (Figure 5A). In line with literature data, CD36 was significantly downregulated in PPAR-γ KO macrophages in comparison to WT macrophages (Figure 5B). Moreover, we could show for the first time that PPAR-γ is required for CD38 expression in macrophages and could confirm that *Cd38* is a target gene for PPAR-γ. To verify the functional significance of PPAR-γ for CD38 expression, we applied ChIP to test the recruitment of the PPAR-γ protein to the putative PPRE located in the intron 1 of the murine *Cd38* gene. Indeed, we could demonstrate the binding of PPAR-γ to the PPRE in WT ER-Hoxb8 macrophages. As expected, no binding was detected in PPAR-γ KO macrophages (Figures 5C,D).

### Deletion of PPAR-γ Leads to Defective Phagocytosis of Latex Beads

**Figure 6:**
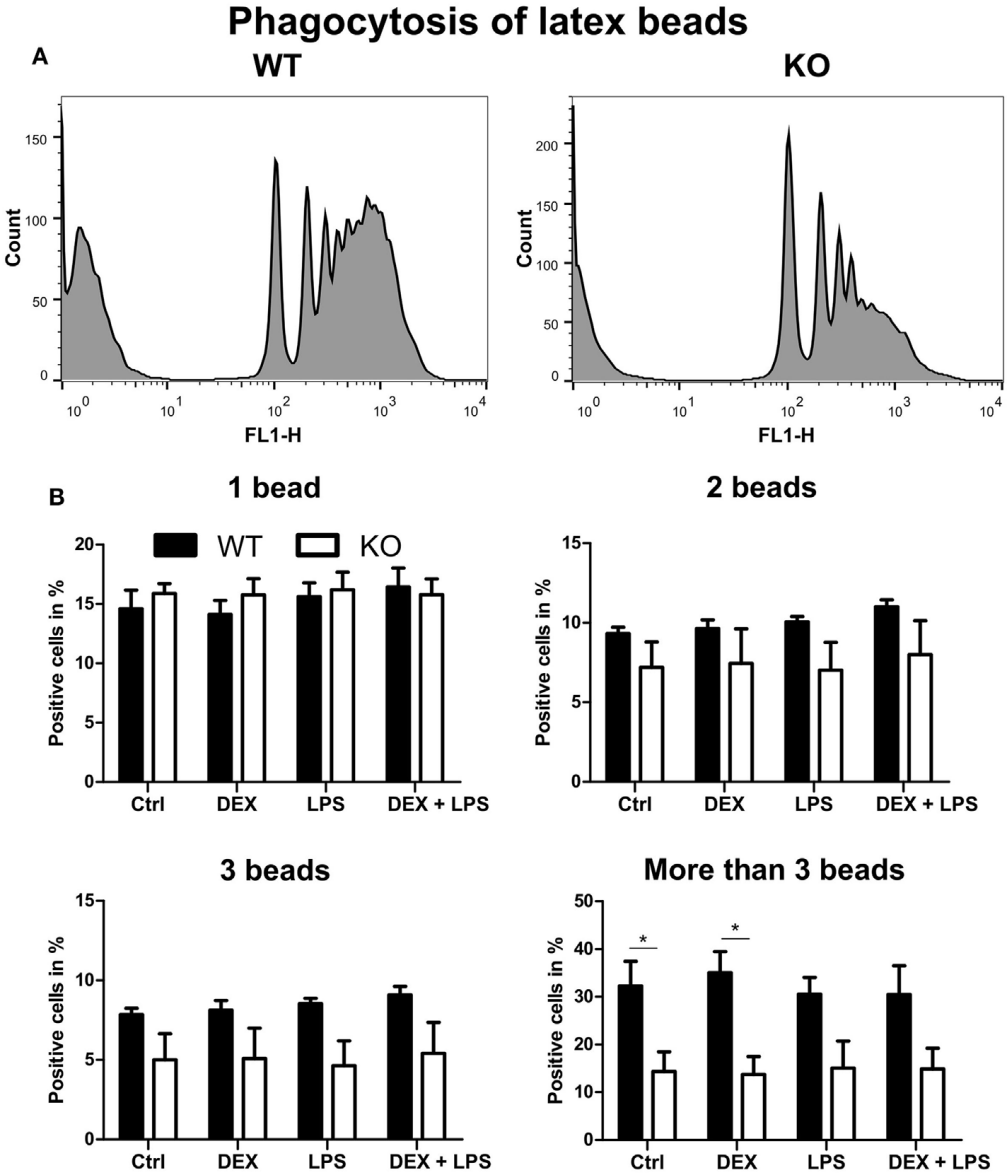
Peroxisome proliferator-activated receptor-γ-deficient macrophages show impaired phagocytosis of latex beads. **(A,B)** ER-Hoxb8 macrophages d5 were treated with dexamethasone (DEX) (100 nM) and/or LPS (1 µg/ml) for 48 h. Subsequently, cells were incubated with fluorescent latex beads for 2 h at a ratio of 10 beads per cells. The phagocytosis was determined in FACS. **(A)** The histograms display results from one representative experiment out of five performed. **(B)** Phagocytosing cells were split into four groups depending on the number of engulfed beads. The bars represent the mean with the SEM of five independent experiments (*p < 0.05) (calculated by using two-way ANOVA).

Since phagocytosis is one of the pivotal functions of macrophages, we next analyzed whether the loss of PPAR-γ, especially in combination with DEX treatment, influenced phagocytosis. WT and PPAR-γ KO macrophages d5 were stimulated with DEX (100 nM) and/or LPS (1 µg/ml) for 48 h. Subsequently, macrophages were incubated with fluorescent latex beads at a ratio of 1:10 for 2 h and analyzed using FACS. We could clearly observe an impaired phagocytic activity of PPAR-γ KO macrophages (Figure 6A). This was especially evident in those cells that phagocytized more than three beads (Figure 6B). A similar trend, although not being significant, was found in LPS- and DEX + LPS-treated macrophages (Figure 6B). In contrast, PPAR-γ-deficient macrophages did not show a reduced ability to phagocytize carboxylate-modified latex beads (data not shown).

### DEX Induces a Pro-Migratory Phenotype in PPAR-γ-Deficient Macrophages

**Figure 7:**
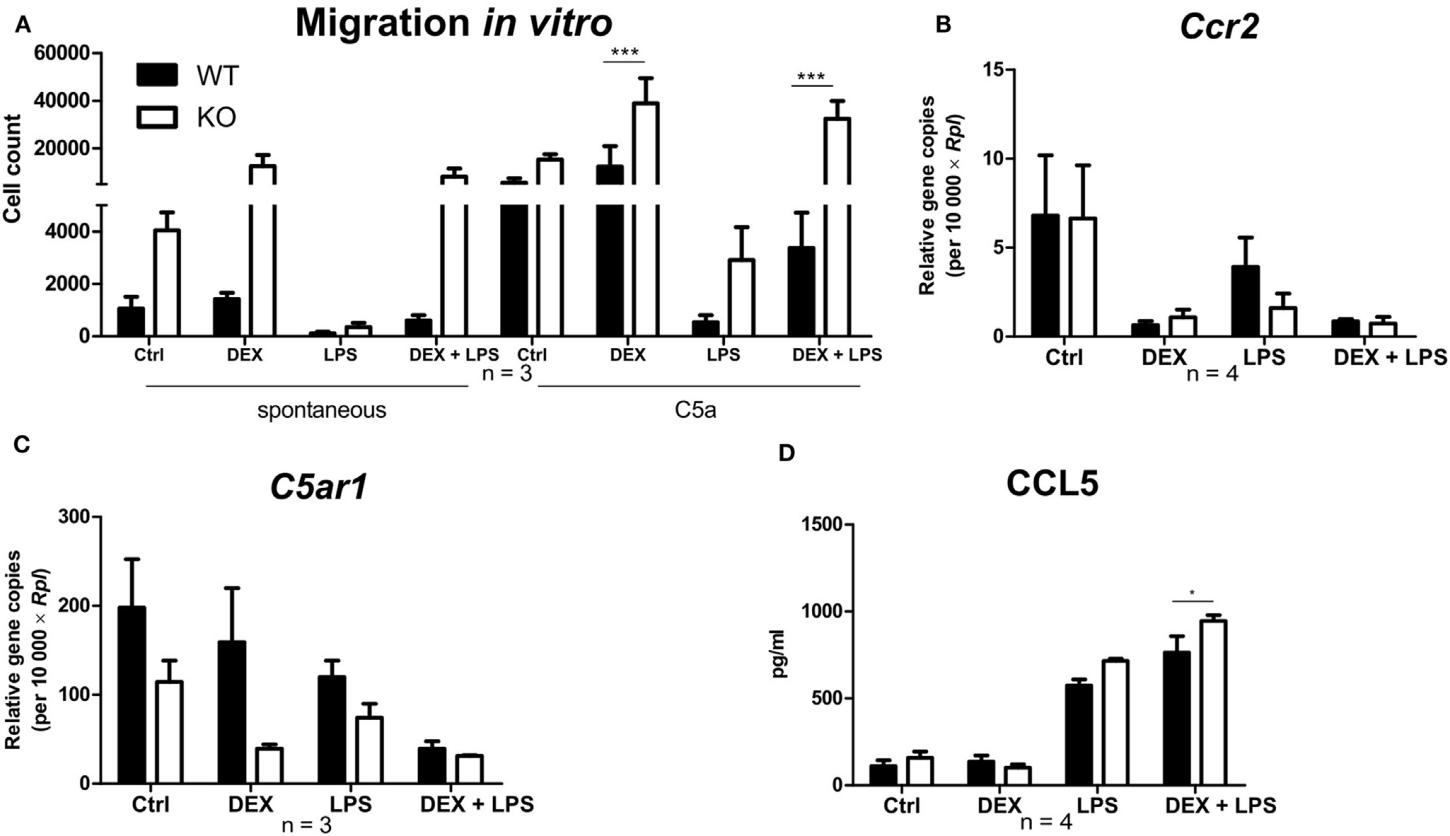
Deletion of peroxisome proliferator-activated receptor-γ promotes a dexamethasone (DEX)-induced increase in migration *in vitro*. **(A–D)** After 5 days of differentiation, ER-Hoxb8 macrophages were stimulated with DEX (100 nM) and/or LPS (1 µg/ml) for 24 h **(B,C)** or 48 h **(A,D)**. **(A)** Spontaneous migration and chemotaxis toward C5a were determined using transwell filters. **(B,C)** Gene expression of the indicated genes was analyzed using qPCR. **(D)** CCL5 secretion to the culture supernatants was determined using CBA. **(A–D)** The bars represent the mean with the SEM of three to four independent experiments (*p < 0.05, ***p < 0.001) (calculated by using two-way ANOVA).

**Figure 8:**
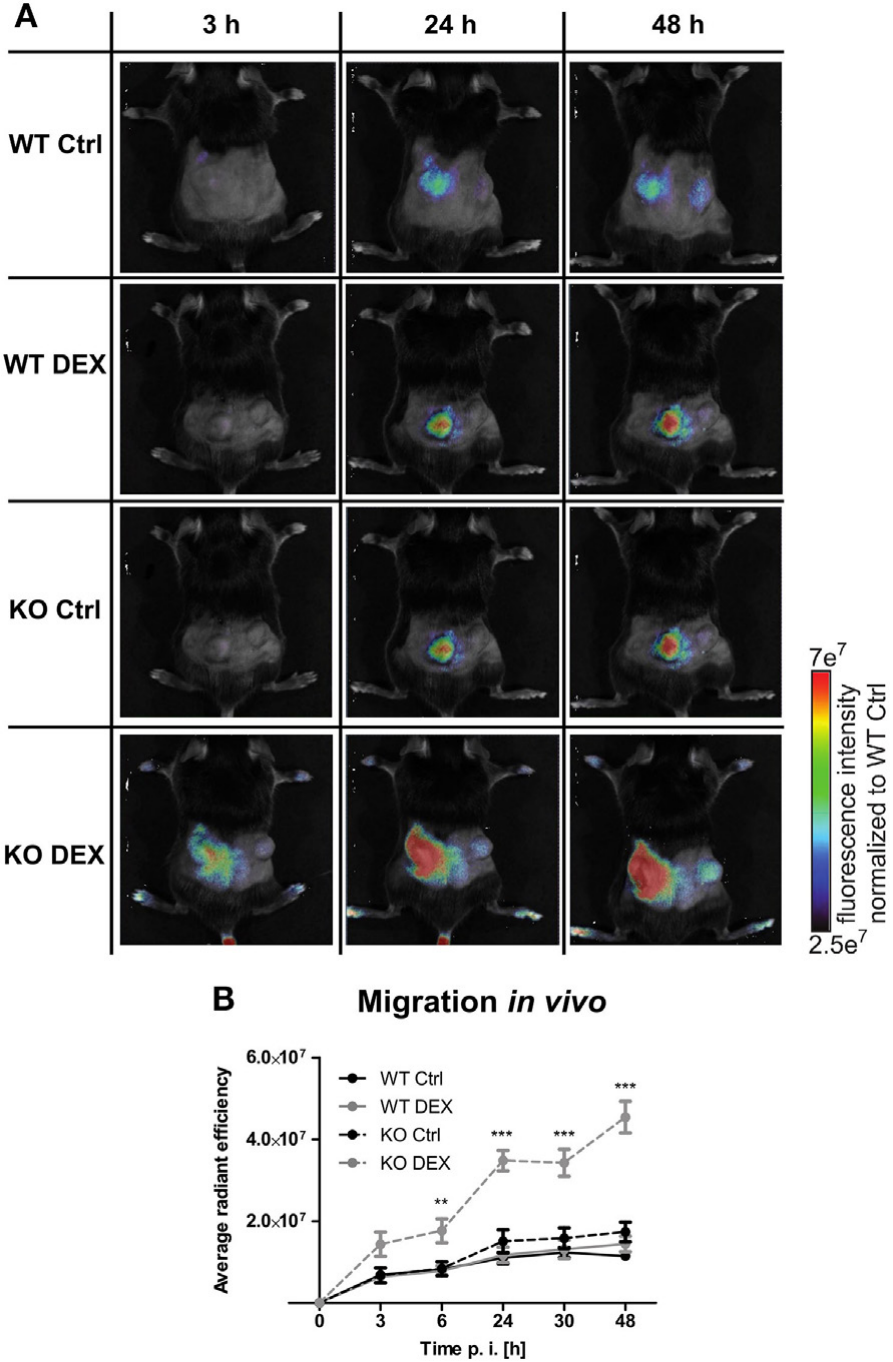
Increased migration in dexamethasone (DEX)-treated peroxisome proliferator-activated receptor-γ-deficient cells *in vivo*. **(A,B)** On day 3 of differentiation, ER-Hoxb8 cells were treated with DEX (100 nM) for 48 h. In parallel, a cutaneous granuloma model (CG) was established by subcutaneous injection of BioGel/LPS (left plug) or BioGel only (right plug) at the dorsal flank region of mice. 24 h later cells were labeled with the fluorescent dye DiD and injected in the tail vein of mice. Migration was tracked at 3, 6, 24, 30, and 48 p.i. by fluorescence reflectance imaging (FRI) using IVIS Spectrum. **(A)** Images show the results from one representative experiment. **(B)** The bars represent the mean with the SEM of five mice of two independent experiments (**p < 0.01, ***p < 0.001) (calculated by using two-way ANOVA).

To investigate the influence of PPAR-γ on macrophage migration, we first performed an *in vitro* migration assay. WT and PPAR-γ KO macrophages were stimulated with DEX and/or LPS for 48 h and subsequently allowed to migrate through transwell filters toward medium (spontaneous migration) or C5a (chemotaxis). We detected a clear tendency of higher spontaneous migration of DEX- and DEX + LPS-treated PPAR-γ-deficient macrophages as compared to WT cells (Figure 7A). In C5a-driven chemotaxis, DEX and DEX + LPS treatment resulted in a significantly augmented chemotaxis of PPAR-γ KO macrophages in comparison to WT cells (Figure 7A). In order to explore the mechanism underlying the enhanced ability of PPAR-γ KO macrophages to spontaneous migration and chemotaxis, we analyzed the expression of several chemokines and their receptors. We found comparable expression of *Ccr2* in WT and PPAR-γ KO macrophages and a tendency of reduced expression of *C5ar1* in PPAR-γ KO cells as compared to WT macrophages (Figures 7B,C). In contrast, CCL5 secretion of PPAR-γ-deficient cells was moderate but significantly upregulated upon DEX + LPS stimulation as compared to DEX + LPS-treated WT macrophages (Figure 7D). To further assess the functional biological relevance of these results, we took advantage of a newly developed *in vivo* cell tracking method (39). Migration of ER-Hoxb8 macrophages was analyzed in a cutaneous granuloma model (CG). WT and PPAR-γ KO ER-Hoxb8 macrophages d3 were stimulated with DEX for 48 h. In parallel, a local granuloma was induced in mice through subcutaneous injection of BioGel/LPS (left dorsal flank) or BioGel only (right dorsal flank). 24 h later, ER-Hoxb8 macrophages were labeled with the fluorescent dye DiD and injected in the tail vein of CG mice. After 3, 6, 24, 30, and 48 h p.i., labeled cell migration was tracked by Fluorescence Reflectance Imaging. We observed no significant differences in *in vivo* migration of untreated WT and PPAR-γ KO macrophages (Figures 8A,B). However, we found that DEX treatment resulted in a significantly increased migration *in vivo* of PPAR-γ KO, but not of WT macrophages. The significant differences were first observed after 6 h p.i. and remained sustained throughout the entire experiment (Figures 8A,B).

## Discussion

Previous studies have revealed that PPAR-γ exerts anti-inflammatory activities toward monocytes and macrophages and can influence their functions as well (14, 17, 30, 32). However, studies using PPAR-γ-deficient mice have provided controversial results (see below). There are several potential pitfalls in analyzing monocyte or macrophage-specific effects in mice. BMCs as source for these primary cells represent a very inhomogeneous cell population. Cultivation with M-CSF results in a more homogeneous, albeit end-stage differentiated macrophage population. The same is true for peritoneal macrophages. In addition, all methods of preparation result in an activation of primary monocytes and macrophages. Last but not least, changes of PPAR-γ expression in other cells or tissues might exhibit bystander effects not intrinsic for monocyte and macrophage functions. Especially the role of PPAR-γ in metabolic processes may have a major indirect input on macrophage functions, since many metabolic products have been shown to modulate inflammatory properties of macrophages (15, 40). In the present study, we took advantage of transiently immortalized PPAR-γ-deficient macrophages to gain a better understanding of the specific role of PPAR-γ in these cells. We established the ER-Hoxb8-system for transient immortalization of bone marrow-derived progenitors of macrophages from *Pparg^fl/fl^* and *LysM-Cre Pparg^fl/fl^* mice. In contrast to primary macrophages, the ER-Hoxb8 system allows the generation of a homogeneous and well-defined population of resting macrophages that are free from secondary effects. Furthermore, ER-Hoxb8 immortalization permits the production of large amounts of cells without time-consuming breeding of mice and without any further purification steps (37). Recently, we could demonstrate that treatment with GC, the most frequently used anti-inflammatory drug, induces a specific anti-inflammatory and pro-resolving phenotype in monocytes (34, 35). However, a role of PPAR-γ on this GC-induced phenotype has not been investigated so far.

Studies regarding the role of PPAR-γ in macrophages differentiation provided contrary results. Whereas some research groups indicated that PPAR-γ promotes macrophage differentiation (14, 41, 42), others found PPAR-γ to be dispensable for this process (13, 15). We could clearly detect the mRNA coding for *Pparg*. The expression of PPAR-γ protein in ER-Hoxb8 progenitor cells was under detection limit but slightly increased during the differentiation to macrophages. However, PPAR-γ KO macrophages revealed clear phenotypic and functional differences as compared to WT cells. This suggests the shaping role of PPAR-γ during the early stages of monocyte/macrophage development despite its low expression levels. In the present study, analyzing ER-Hoxb8 murine macrophages, we clearly observed a delayed differentiation kinetic of PPAR-γ-deficient bone marrow-derived monocytes into macrophages, characterized by reduced F4/80 but increased Ly6C expression in early phases of differentiation as compared to WT macrophages. It has been known for a long time that F4/80 expression increases progressively during maturation of macrophages (43). Reduced F4/80 expression in PPAR-γ-deficient cells in early phases of differentiation corresponded with an immature macrophage morphology in this phase. In contrast, comparable F4/80 expression in WT and PPAR-γ-KO cells on day 5 of differentiation indicated a catch up of PPAR-γ KO cells. Since monocytes migrating to sites of inflammation were found to express higher levels of Ly6C than patrolling monocytes (44), the increased Ly6C expression is consistent with the pro-inflammatory and pro-migratory phenotype that we observed in PPAR-γ-deficient macrophages.

It has been demonstrated that PPAR-γ agonists inhibit the production of pro-inflammatory cytokines, such as TNF-α, IL-1β, and IL-6 (17, 45). However, it has also been shown that the inhibitory activity of the PPAR-γ antagonist 15d-PGJ2 could not be blocked by adding PPAR-γ-agonists (19) and that 15d-PGJ2 used at high concentrations inhibited the iNOS promoter even in PPAR-γ-deficient cells (20). These controversies clearly show the need to define the role of PPAR-γ in inflammation by using a macrophage-specific PPAR-γ KO system independent from other cell populations. Studies using primary PPAR-γ KO macrophages revealed increased expression of pro-inflammatory cytokines in PPAR-γ-deficient macrophages (5, 46). In line with these findings, we could now show that long-term stimulation with LPS induced a distinct pro-inflammatory phenotype in PPAR-γ KO ER-Hoxb8 macrophages, characterized by an increase of pro-inflammatory cytokines TNF-α, IL-1β, and IL-6 and a reduction of anti-inflammatory cytokine IL-10. Interestingly, the pro-inflammatory phenotype was not observed during the early response to LPS. There are mixed opinions regarding the effect of PPAR-γ on the expression of the LPS co-receptor CD14 in macrophages (13, 28). However, we found no major differences between WT and PPAR-γ-deficient cells regarding the receptors for LPS (CD14 and TLR4), indicating that down-stream signaling or gene expression is modulated by PPAR-γ. Since PPAR-γ has been described to be important for resolving inflammation and maintaining homeostasis, the pro-inflammatory phenotype observed in PPAR-γ KO macrophages might be a consequence of altered or missing resolution of inflammation (31). Macrophages that are missing PPAR-γ seem to tend to a long-lasting and sustained immune response to LPS and/or are not able in course of inflammation to re-program toward a pro-resolving phenotype.

So far, only few studies have analyzed the influence of PPAR-γ on ROS production. It has been reported that PPAR-γ-deficient macrophages display enhanced ROS production in response to infection with *L. monocytogenes* as compared to WT macrophages (32). We did not detect substantial differences in ROS production between WT and PPAR-γ KO macrophages. In addition, it has been demonstrated that PPAR-γ agonists inhibit NO production (6). However, other groups have provided contrary results and have shown that PPAR-γ agonists could also reduce *Nos2* expression in PPAR-γ-deficient macrophages, suggesting an involvement of a PPAR-γ-independent mechanism (7, 20). In this study, we observed diminished NO production in ER-Hoxb8 PPAR-γ-deficient macrophages, indicating that PPAR-γ is required for a sufficient NO production during macrophage differentiation.

In the present study, we focused on the role of PPAR-γ in GC-induced effects in macrophages. In preadipocytes (47) and colonic tissues (48), DEX treatment led to an increased PPAR-γ expression. However, the effects of GC on PPAR-γ expression in macrophages have not previously been studied. We could demonstrate that PPAR-γ protein expression was significantly increased in ER-Hoxb8 macrophages upon DEX and DEX + LPS stimulation. However, there were no significant differences in DEX-mediated suppression of LPS-induced pro-inflammatory (TNF-α, IL-1β, IL-6, and IL-12) and anti-inflammatory (IL-10) cytokines in WT and PPAR-γ KO ER-Hoxb8 macrophages, implicating that major DEX-mediated effects on the inflammatory cytokine response are PPAR-γ-independent.

To our knowledge, the role of PPAR-γ in regulating CD86 expression in macrophages has not yet been analyzed. We could now demonstrate that loss of PPAR-γ led to a diminished CD86 expression in macrophages. Since CD86 is a crucial T cell costimulatory molecule, PPAR-γ-deficient macrophages are expected to be less efficient in activating T cells. Our finding is in accordance with studies showing that activation of PPAR-γ raised CD86 expression in DCs (12, 49, 50).

It has been reported that ablation of PPAR-γ in macrophages resulted in a defective phagocytosis (30, 31). Consistent with these findings, we observed impaired phagocytosis in PPAR-γ-deficient macrophages. In addition, it has been reported that impaired clearance of apoptotic cells in PPAR-γ-deficient macrophages resulted in a delayed skin wound healing and that PPAR-γ agonists could accelerate this process in WT, but not in PPAR-γ-deficient mice (31). It has been shown that GC treatment induced a pro-resolving phenotype in macrophages, partially by exhibiting a positive effect on phagocytosis (51, 52). In addition, GC-induced augmentation of phagocytosis of apoptotic cells in human macrophages could be reversed by costimulation with DEX and a PPAR-γ antagonist (30). However, in our murine system, we could neither detect a GC-induced increase in phagocytosis in WT nor in PPAR-γ KO ER-Hoxb8 macrophages. CD36, a known target of PPAR-γ, plays an essential role in the uptake of apoptotic cells (14, 15, 49, 53, 54). Accordingly, we observed a reduction of CD36 expression in PPAR-γ-deficient macrophages. In our study, the deletion of PPAR-γ also led to a significant reduction of CD38 expression, which has recently been described to be a target gene of PPAR-γ in adipocytes (38). We could also demonstrate that CD38 expression in macrophages depends on PPAR-γ and using ChIP we could confirm that CD38 is a direct target gene of PPAR-γ. CD38 is a multifunctional enzyme, which has gained increasing attention in the last years. Interestingly, CD38 has been suggested to regulate FcγR-mediated phagocytosis and alter migration patterns of inflammatory monocytes to sites of inflammation (55, 56).

In line with the latter finding, we found augmented migration in PPAR-γ-deficient macrophages (32, 57, 58). This pro-migratory phenotype of PPAR-γ KO cells corresponded well to their increased Ly6C expression, since Ly6C^high^ monocytes are known to migrate to sites of inflammation, while Ly6C^low^ monocytes patrol the blood vasculature (59). It has been reported that GC treatment augments both spontaneous migration as well as chemotaxis in human monocytes (34). Interestingly, we detected significantly increased *in vitro* migration in GC-treated PPAR-γ-deficient, but not in WT macrophages. The regulatory role of PPAR-γ in GC-induced modulation of migratory capability of macrophages is a new feature of PPAR-γ. To further assess the functional biological relevance of these results, we took advantage of a newly developed *in vivo* cell tracking method in a cutaneous granuloma model (39). Indeed, we could show that PPAR-γ KO but not WT macrophages treated with GC showed dramatically enhanced recruitment to the site of inflammation. To better understand the underlying mechanism of enhanced migration in PPAR-γ-deficient macrophages, we analyzed the expression of several chemokines and their receptors. It has been reported that CCR2 expression is increased in PPAR-γ-deficient myeloid cells (32). Moreover, CCR2 WT BMCs were recruited more efficiently in early phases after induction of peritonitis than CCR2 KO cells (60). However, in the present study, there were no significant differences in CCR2 expression between WT and PPAR-γ KO ER-Hoxb8 cells. Inhibition of CCL5 by PPAR-γ agonists was described for endometrial stroma cells and DCs (49, 61). Consistently, we found augmented CCL5 secretion in PPAR-γ-deficient macrophages upon simultaneous LPS and DEX stimulation, indicating that an increased CCL5 secretion in PPAR-γ-deficient macrophages could contribute to their enhanced migration in response to GC treatment.

In conclusion, we could show that the loss of PPAR-γ in macrophages leads to altered differentiation kinetics. Moreover, we demonstrated that PPAR-γ deficiency results in a pro-inflammatory phenotype in macrophages and that PPAR-γ shapes macrophage functions, such as phagocytosis and migration. Finally, we identified a functional link between GC and PPAR-γ. First, we could show that GC enhanced PPAR-γ expression both in resting and pro-inflammatory (LPS-treated) macrophages. Second, we demonstrated for the first time that GC treatment significantly induced *in vitro* and *in vivo* migration of macrophages. This was observed only in the absence of PPAR-γ, implicating a negative role of PPAR-γ in macrophage migration. Surprisingly, we observed no differences between WT and PPAR-γ KO macrophages in GC-mediated suppression of LPS-induced pro-inflammatory cytokine production. Our data demonstrate that analysis of stem cell-derived monocytes and macrophages is a reliable system to analyze intrinsic effects of specific knockouts in the monocyte and macrophage lineage, which are partly hidden by complex interactions or are even artificially induced by bystander cells.

## Ethics Statement

All animal experiments were carried out in accordance with the German animal protection law (TierSchG). The protocol was approved by the government authorities (Landesamt für Natur, Umwelt und Verbraucherschutz Nordrhein-Westfalen).

## Author Contributions

MH performed large parts of the research and wrote the manuscript. SG performed the in vivo migration assay and was involved in writing the manuscript. S-JL performed chromatin immunoprecipitation and was involved in writing the manuscript. LF-R and AR performed revision experiments. LK was involved in establishment of the PPAR-γ knock-out cell lines. SH and MS designed and supervised the in vivo migration assay. JR designed the experiments, was involved in writing the manuscript and supervised the project. KB-K performed ER-Hoxb8 immortalization, designed the experiments, wrote the manuscript and supervised the project.

## Conflict of Interest Statement

The authors declare that the research was conducted in the absence of any commercial or financial relationships that could be construed as a potential conflict of interest. The reviewer TB and the handling Editor declared their shared affiliation.
