# Supplementary material for: Peroxisome Proliferator-Activated Receptor-γ Modulates the Response of Macrophages to Lipopolysaccharide and Glucocorticoids
Source: Front Immunol. 2018 May 8;9:893. doi: 10.3389/fimmu.2018.00893 (PMC5949563; doi:10.3389/fimmu.2018.00893)
Supplement: Supplementary file 1 [file Data_Sheet_1.docx]

Supplementary Material

PPAR-γ Modulates the Response of Macrophages to Lipopolysaccharide and Glucocorticoids

Michael Heming, Sandra Gran, Saskia-L. Jauch, Lena Fischer-Riepe, Antonella Russo, Luisa Klotz, Sven Hermann, Michael Schäfers, Johannes Roth and Katarzyna Barczyk-Kahlert*

*** Correspondence:** Corresponding Author: Katarzyna Barczyk-Kahlert bar@uni-muenster.de

# Supplementary Figures

##
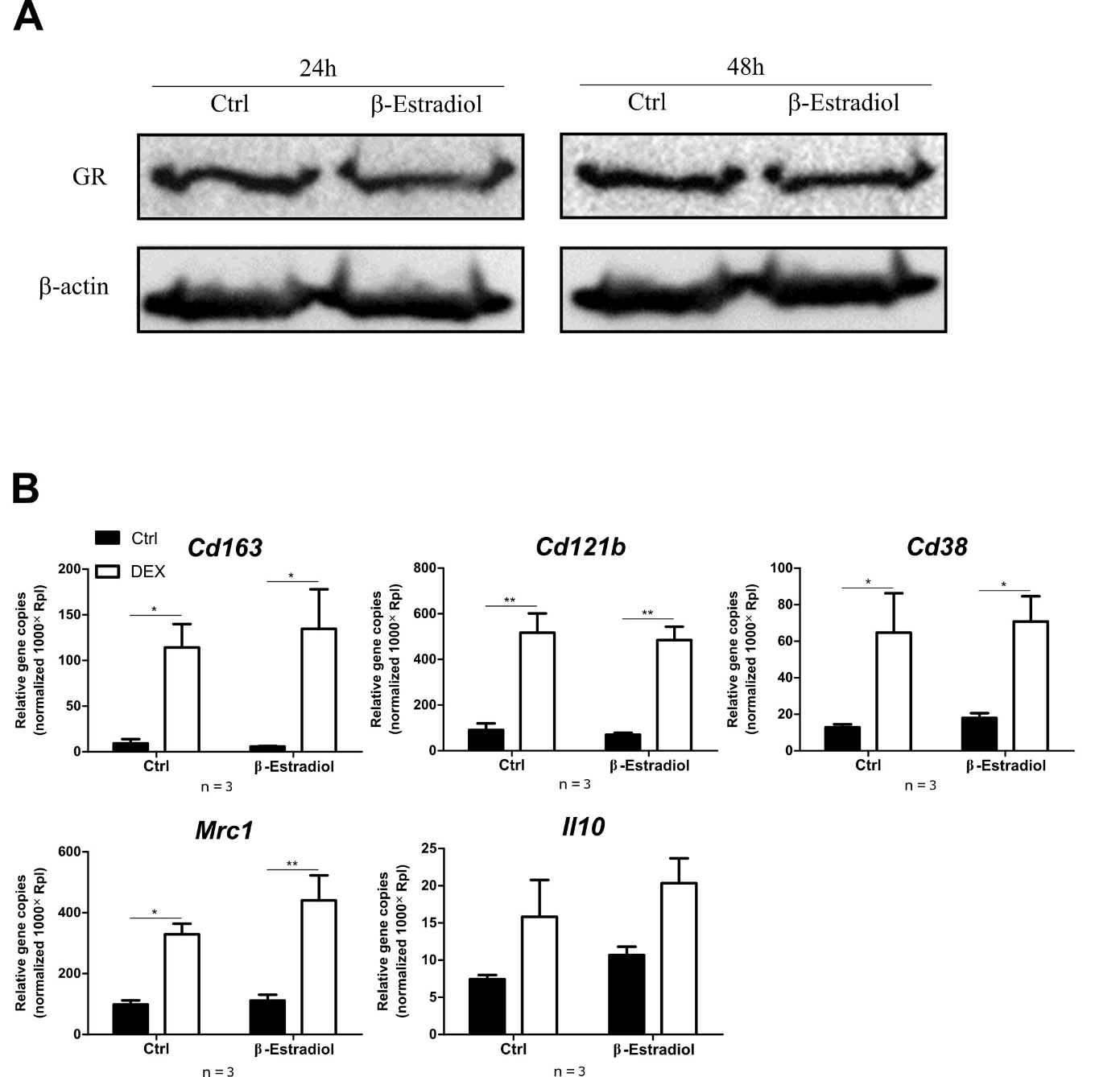


**Supplemental Figure 1.** β-estradiol does not influence the GC sensitivity. Bone marrow-derived monocytes were pretreated in the presence of β-estradiol for 24 h **(A,B)** and 48 h **(A)**. **(A)** cells were analyzed for the expression of glucocorticoid receptor in Western blot. β-Actin was used as a loading control. **(B)** Control and β-estradiol treated bone marrow-derived monocytes were stimulated with DEX for 24 h and subsequently gene expression of the indicated genes was analyzed using qPCR. The bars represent the mean with the SEM of 3 independent experiments. * *p* < 0.05, ** *p* < 0.01 (calculated by using two-way ANOVA).
